# Altered Trek-1 Function in Sortilin Deficient Mice Results in Decreased Depressive-Like Behavior

**DOI:** 10.3389/fphar.2018.00863

**Published:** 2018-08-06

**Authors:** Sébastien Moreno, Christelle M. Devader, Mariel Pietri, Marc Borsotto, Catherine Heurteaux, Jean Mazella

**Affiliations:** Centre National de la Recherche Scientifique, Institut de Pharmacologie Moléculaire et Cellulaire, UMR 7275, Université Côte d’Azur, Valbonne, France

**Keywords:** depression, sortilin, TREK-1, behavior, electrophysiology

## Abstract

The background potassium channel TREK-1 has been shown to be a potent target for depression treatment. Indeed, deletion of this channel in mice resulted in a depression resistant phenotype. The association of TREK-1 with the sorting protein sortilin prompted us to investigate the behavior of mice deleted from the gene encoding sortilin (*Sort1*^−/−^). To characterize the consequences of sortilin deletion on TREK-1 activity, we combined behavioral, electrophysiological and biochemical approaches performed *in vivo* and *in vitro*. Analyses of *Sort1*^−/−^ mice revealed that they display: (1) a corticosterone-independent anxiety-like behavior, (2) a resistance to depression as demonstrated by several behavioral tests, and (3) an increased activity of dorsal raphe nucleus neurons. All these properties were associated with TREK-1 action deficiency consequently to a decrease of its cell surface expression and to the modification of its electrophysiological activity. An increase of BDNF expression through activation of the furin-dependent constitutive pathway as well as an increase of the activated BDNF receptor TrkB were in agreement with the decrease of depressive-like behavior of *Sort1*^−/−^ mice. Our results demonstrate that the TREK-1 expression and function are altered in the absence of sortilin confirming the importance of this channel in the regulation on the mood as a crucial target to treat depression.

## Introduction

The origins of mood disorders result from a complex cross-talk between multiple genes in relation with environmental and developmental epigenetic components. Our understanding of the biological mechanisms of such diseases is therefore limited. We only accumulate informations about the roles of some crucial central and peripheral mediators such as corticosterone, 5-HT and BDNF. Briefly, depressive patients show an increase of circulating corticosterone, a modification of 5-HT content and a marked decrease of both cerebral and peripheral circulating BDNF levels. These mediators belong to the epigenetics and gene expression marker family of major depressive disorder and AD response (for review see Fabbri et al., 2017). At the level of animal models, transgenic mice allowed to identify some new targets.

Phenotyping mice in which a gene has been deleted may appear relatively easy in terms of preliminary observations for detecting a major health problem or a motor defect. A series of physical parameters including body weight and temperature, home cage locomotion, grooming, nesting and sleeping, neurological reflexes can be tested before starting behavioral tasks related to anxiety and mood disorders (Crawley, 1999). In the field of depression and anxiety-related disorders, the background potassium channel TREK-1 (Twik related potassium channel 1) has been one of the first targets for which deletion of its gene (*Kcnk2*^−/−^) in mice resulted in a depression-resistant phenotype highlighted by behavioral tests (Heurteaux et al., 2006). Interestingly, in several behavior tests, *Kcnk2*^−/−^ mice behave, similarly, to naive mice treated with ADs like fluoxetine. In addition, an increase in firing rate of serotoninergic neurons from the DRN is observed, increasing the 5-HT transmission being one of the main characteristics of AD action (Heurteaux et al., 2006). Taken together, results obtained from *Kcnk2*^−/−^ mice led to the hypothesis that the search of selective blockers of the TREK-1 channel could lead to a new type of AD. More recently, the discovery of spadin, which is a sortilin-derived peptide acting as a potent AD and specifically targeting TREK-1 channels, supported this hypothesis (Mazella et al., 2010). Interestingly, in addition to the AD properties of spadin, we have demonstrated the existence of a protein complex between the TREK-1 channel and sortilin. The expression of the TREK-1 channel at the PM of COS-7 cells is increased when the channel is co-expressed with sortilin. Consequently, because sortilin is a crucial partner in the sorting of several factors and enzymes (Lefrancois et al., 2003; Kandror and Pilch, 2011), we analyzed in details the depression-related phenotype of mice in which the gene encoding sortilin (*Sort1*) has been inactivated, with a focus on the expression of its partner TREK-1 and the subsequent consequences on the TREK-1 function.

To date, inactivation of *Sort1* gene demonstrated that sortilin is involved in the proNGF (precursor of nerve growth factor)-induced neuronal cell death when associated with p75NTR (p75 neurotrophin receptor) (Nykjaer et al., 2004) as well as in the binding and internalization of the fronto-demential protein progranulin (Hu et al., 2010). More recently a controversial role of sortilin in the regulation of cholesterol metabolism has been described (Kjolby et al., 2010; Musunuru et al., 2010), and largely commented in the literature (Dube et al., 2011; Tall and Ai, 2011). Sortilin [also called NTSR3] belongs to the neurotensinergic system (Mazella et al., 1998). A recent study showed that the lack of sortilin expression leads to the increase in brain of both NT and NTSR2 receptors. These *Sort1*^−/−^ mice are less sensitive to thermal and chemical nociception (Devader et al., 2016).

Sortilin is subjected to membrane protease-induced shedding leading to the release of a soluble form (Navarro et al., 2002). Interestingly, increased serum concentrations of soluble sortilin have been detected in patients suffering from major depression when compared to control healthy subjects (Buttenschon et al., 2015). The higher level of soluble sortilin in depressive patients is correlated with an increase of BDNF and VEGF levels, indicating that the circulating soluble sortilin could be a new candidate as a biomarker of depression state. However, a more recent study investigating the serum level of sortilin in response to AD treatment revealed that the depression score and response to treatment were not predicted by sortilin level (Buttenschon et al., 2017), then invalidating the former hypothesis.

Because sortilin and TREK-1 have been shown to be associated at the cellular and molecular levels (Mazella et al., 2010) and because sortilin plays an important role in the sorting of numerous proteins we hypothesized that the absence of sortilin could result in modifications of TREK-1 expression, its cellular location and its functions. On another hand, since TREK-1 was described to be a potent target in depression (Heurteaux et al., 2006), the aim of the present work was to analyze using electrophysiological, biochemical, and behavioral approaches, the phenotype of *Sort1*^−/−^ mice regarding antidepressive-like behavior.

## Materials and Methods

### Animals

The NTSR3/sortilin homozygous KO mice (*Sort1*^−/−^*)* were generated by the Morales’s laboratory by incorporation of a GFP cassette after exon 1 (Zeng et al., 2009) and controls were C57Bl/6J male mice from Janvier Labs (Saint-Berthevin, France). Both strains were of the same C57Bl/6J genetic background. *Sort1*^−/−^ mice were used after more than 10 generations and then considered with a stable genetic background. The local Ethics Committee (CIEPAL) (protocol number 00893.02) approved experimental procedures and animal cares are in accordance with the policies on the care and use of laboratory animals of European Community legislation 2010/63/EU. Animal studies were conducted in compliance with the ARRIVE guidelines (Kilkenny et al., 2011; McGrath and Lilley, 2015). Adult male (8–10 weeks old) mice were housed under controlled laboratory conditions according to the FELASA guidelines and recommendations; 6 mice/cage with a 12 h dark–light cycle, a temperature of 21 ± 2°C, and a 40–60% humidity. They have free access to standard rodent diet and tap water.

### Sources of Reagents and Equipment

The ELISA kit to measure corticosterone was from Enzo Life Science. Rabbit polyclonal antibodies anti-TREK-1 and anti-proBDNF were from Alomone Labs (Israel). Rabbit polyclonal antibodies anti-furin and anti-NaKATPase, the mouse monoclonal anti-beta-actin and goat anti-TGN38 and anti-DCX antibodies were from SantaCruz Technologies (United States). Rabbit polyclonal antibodies anti-TrkB and anti-phospho-(Tyr 705)-TrkB were from Signalway Antibody (United States). Rabbit polyclonal antibodies against BDNF and plasminogen were from GeneTex (United States). Mouse monoclonal anti-BrdU antibody was from Becton Dickinson (United States). Anti-rabbit, anti-mouse, and anti-goat secondary antibodies were from Cell Signaling Technologies. HRP substrate (Immobilon Forte, Millipore), Nitrocellulose (BioTrace NT, Life Sciences), SDS-PAGE gels and Mini Protean apparatus were from Biorad. BrdU was from Interchim (France). Neurobasal medium, B-27 and Penicillin-Streptomycin were from Thermo Fisher Scientific. Poly-D-Lysine was from Sigma-Aldrich. Patch clamp was performed on a pClamp 8.2 apparatus (Molecular Devices)

### Primary Culture of Cortical Neurons

Primary cortical neurons were isolated from 14-day-old mouse embryo cortice (E14) from *Sort1*^−/−^ and control females. Cells were mechanically dissociated and seeded in 35 mm diameter dishes previously treated with Poly-D-Lysine and maintained in culture in a Neurobasal/B27 medium for 10–14 days.

### Data and Statistical Analysis

#### Group Sizes

The group sizes provided for the following experiments were variable due to the difficulties to regularly obtains the same number of adult males (8–10 weeks old) house breeded animals (i.e., *Sort1*^−/−^ mice). For each kind of experiment, we adapted the number of WT mice to the number of available *Sort1*^−/−^ mice.

Each animal was used once and the total of 218 male WT and *Sort1*^−/−^ mice were used for these studies. Behavioral experiments were performed with naïve mice for all used tests. Mice were isolated 30 min in neutral room before tests. The experimenter was blind to randomized experimental groups. Randomization was performed using the TST software (Bioseb, France).

Behavioral tests used in this work have been validated for mouse behavioral phenotypes related to neuropsychiatric disorders, such as depression or anxiety (for review, see Samsom and Wong, 2015). Group sizes for behavioral, biochemical, and electrophysiological experiments were equal with the exception for firing rate recordings for which measurements were recorded from 36 5-HT neurons for WT and 26 neurons for *Sort1*^−/−^ mice. These *in vivo* experiments were performed on five animals for each group and the number of recorded neurons from each animal varied depending on the position of electrodes.

For Western blot analyses, results were normalized using either an intracellular protein (actin) or a protein specific for a given intracellular compartment for sub-cellular fractionation experiments.

All data (displayed as mean ± SEM) were analyzed using Prism 6-2 Software (GraphPad, San Diego, CA, United States). For the comparison of two groups, Mann–Whitney test was used except for measurement of the DRN firing rates for which Student’s *t*-test was used. All remaining data were compared using a two-way ANOVA with a Tukey’s multiple comparison test. The level of significance was set at *p* < 0.05. The data and statistical analysis comply with the recommendations on experimental design and analysis in pharmacology (Curtis et al., 2015).

### Behavioral Tests

#### Forced Swimming Test (FST)

Forced swimming test was performed according to the procedure initially described (Porsolt et al., 1977). In order to validate the test and to analyze whether the effects observed can be additive, in some cases, spadin (i.p. 100 μl of 10^−6^ M) or saline were injected 30 min before the test. Each animal was placed in a cylinder (height 30 cm, diameter 15 cm) filled with 15 cm water at 22 ± 1°C with no escape possibility during 6 min. The period of immobility was measured only during the last 4 min of the trial. We considered an immobile mouse when it only remained floating with slight movements to maintain head above surface.

#### Novelty Suppressed Feeding (NSF)

Mice were deprived from food for 24 h. On test day, mice were placed in a highly brightly lit box (45 cm × 45 cm × 20 cm), with floor covered with wood chip bedding, during 10 min. At the center of the box, a food pellet was placed on a white platform. The latency for the animal to eat the pellet was measured as previously described (Santarelli et al., 2003).

#### Tail Suspension Test (TST)

Mice were suspended by the tail by using a piece of adhesive tape. After “agitation” or “escape-like” behavior, mice adopted an immobile posture, suggested to mirror a state of depression. We recorded the immobility time during a 6 min test session according to Cryan et al. (2005).

#### Marble Burying

Burying object relates to a natural defense mechanism that occurs in mice under stress condition or anxiety state. Marble burying is able to detect anxiety and obsessive compulsive disorders-related phenotypes (Deacon, 2006). In response to novel bedding/environment mice exhibit digging behavior. Mice were placed during 30 min in a cage filled with approximately 5 cm deep with wood chip bedding and a regular pattern of 13 glass marbles disposed on the surface, evenly spaced, each about 4 cm apart. At the end of the time, buried marbles were counted (2/3 minimum of depth). Seventy-five percent of buried marbles was a typical score for naïve C57BL/6.

#### Elevated Plus Maze

Elevated Plus Maze allows to define an anxiety response in rodents (Gross et al., 2002). The apparatus consisted of central platform (5 cm × 5 cm), two open arms and two closed arms across from each other and perpendicular, with the same size (45 cm × 5 cm) and 15 cm wall height for the closer arms. The apparatus was placed at 45 cm height above the floor. Mice were placed in the central platform facing one open arm and were allowed to freely move during 10 min. During this period, number of entries and time spent in both arms were measured. To define an anxiety response, the time and number of entries in open arms were evaluated in relation to whole time spent or the total entries.

#### Light Dark

Mice were placed in a box divided into two compartments by a black partition with a small opening that allows mouse to move from one compartment to the other (Welch et al., 2007). One compartment, corresponding to one-third of the surface area, was made of white plastic and was brightly illuminated. The adjoining smaller compartment was black and dark. Mice were placed in the white compartment and allowed to move freely between the two chambers for 5 min. Time spent in the white chamber, and latency to the first transition were recorded. Mice tended to avoid the white compartment. The measures of exploration in this area were used as experimental indices of anxiety.

### Measurement of Serum Corticosterone Levels

Corticosterone concentration present in the serum from WT and *Sort1*^−/−^ mice was determined by blood punctures made between 9 and 10 a.m. using the ELISA kit from Enzo Life Science (Cat. No. ADI-900-097) according to the manufacturer recommendations. The sensitivity of the detection was between 32 and 20,000 pg/ml and was species independent.

### Whole Cell Patch Clamp Recordings and Membrane Potential Measurements

Electrophysiological experiments were performed on primary cortical neurons seeded at a density of 100,000 cells/35 mm dish after 10 days of culture. Membrane potential was recorded in whole cell configuration (Hamill et al., 1981) in current clamp mode (no current is injected, I = 0). Each membrane potential was evaluated by using a RK 400 patch clamp amplifier (Axon Instruments, United States), low-pass filtered at 3 kHz and digitized at 10 kHz using a 12-bit analog-to-digital converter Digidata (1322 series, Axon Instruments, United States). Patch clamp pipettes were pulled using vertical puller (PC-10, Narishige) from borosilicate glass capillaries and had a resistance of 10 MΩ. The bath solution contained (in mM) 150 NaCl, 5 KCl, 3 MgCl_2_, 1 CaCl_2_, and 10 HEPES adjusted to pH 7.4 with NaOH. The pipette solution contained (in mM) 155 KCl, 3 MgCl_2_, 5 EGTA, and 10 HEPES adjusted to pH 7.2 with KOH. All experiments were performed at room temperature (21–22°C). Data acquisition was carried out using a microcomputer (Dell Pentium) with commercial software and hardware (pClamp 8.2). All values of membrane potentials are expressed in mV as mean ± standard error of the mean (SEM).

### Sub-cellular Fractionation

In order to quantify the amount of TREK-1 expressed at the PM and intracellularly, we performed sub-cellular fractionation from brain homogenates. PMs were prepared from brain homogenates of WT or *Sort1*^−/−^ mice according to the protocol previously described (Clancy and Czech, 1990). Briefly, brain from WT or *Sort1*^−/−^ mice were collected in Solution A (250 mM sucrose, 20 mM HEPES, 1 mM EDTA and 1 mM PMSF at pH 7.4), homogenized with a Teflon pestle, and centrifuged at 16,000 × *g* for 20 min to yield Pellet 1 and Supernatant 1. Pellet 1 was resuspended in Solution B (20 mM HEPES and 1 mM EDTA at pH 7.4) using a Dounce homogenizer, loaded on a sucrose solution (1.12 M Sucrose in Buffer B), and centrifuged at 100,000 × *g* for 60 min to yield Pellet 2 and Supernatant 2. Pellet 2 representing the nuclear/mitochondrial fraction was discarded. Supernatant 2 was collected at the interface between the sucrose solution and Buffer B and was resuspended in Buffer B and centrifuged at 30,000 × *g* for 30 min to yield Pellet 3; pellet 3 was resuspended in Buffer B and represented the PM fraction. Meanwhile, Supernatant 1 was centrifuged at 30,000 × *g* for 30 min to produce Pellet 4 and Supernatant 3. Pellet 4 was resuspended in Buffer B and represented the HDM fraction. Supernatant 3 was centrifuged at 200,000 × *g* for 90 min to yield Pellet 5 resuspended in Buffer B and represented the LDM fraction. HDM and LDM were pooled for Western blot analyses 30 μg of crude homogenates, purified PMs and H/LDM were submitted to Western blot analysis using the rabbit polyclonal antibody against Anti-K2P2.1 (TREK-1) (1:500) (Alomone Labs, Israel). Proteins detected with this antibody were normalized using antibodies specific for each intracellular compartment (NaKATPase for PMs, TGN38 for H/LDM and beta-actin for total extracts) from SantaCruz Technologies (United States).

### Western Blot

Mouse brains were dissected on ice and then homogenized in a solubilization buffer containing 20 mM HEPES (pH:7.4), 1 mM EDTA, 1 mM PMSF, 250 mM sucrose, and protease inhibitor cocktail using a polytron at the lowest speed. The homogenates were centrifuged 20 min at 100,000 × *g* at 4°C. Supernatants were resupended in 20 mM HEPES (pH: 7.4), 1 mM EDTA and stored at −20°C until further use. Solubilized proteins were loaded at 50 μg in SDS buffer, separated on 10% SDS PAGE gels and then transferred to a nitrocellulose membrane.

Membranes were incubated with rabbit polyclonal Anti-BDNF (1:1000) (GeneTex, United States), Anti-Phospho(Tyr705)TrkB (1:500), Anti-TrkB (1:1000) (Signalway Antibody, United States), Anti-K2P2.1 (TREK-1) (1:500) and anti-ProBDNF (1:400) (Alomone Labs, Israel), Anti-Furin (1:1000) (SantaCruz Technologies, United States), Anti-Phospho(Ser133) CREB (1:500) and mouse monoclonal Anti-CREB (1:1000) (CST, United States), mouse monoclonal Anti-beta-Actin (1:5000) (SantaCruz Technologies, United States) over night at 4°C. Afterwards, membranes were incubed 30 min with secondary antibody (related to species of first antibody) coupled HRP. Protein bands were revealed, images were acquired with FX Fusion (Vilber) and analyzed with ImageJ (US NIH, Bethesda, MD, United States) (Schneider et al., 2012).

### Measurement of Hippocampal Neurogenesis

Mice were treated either with saline or with spadin (i.p. 100 μl of 10^−6^ M) once daily for 4 days and the fourth day BrdU was also injected (120 mg per kg of body weight in a 300 μl bolus). Twenty-four hours after the BrdU injection, mice were euthanized and transcardially perfused with 4% cold paraformaldehyde. Serial brain sections were cut (40 μm) throughout the entire hippocampus on a vibratome (Leica). Every sixth sections, slices were processed for BrdU and DCX immunohistochemistry, as previously described (Heurteaux et al., 2006). For each immunodetection, slices were first incubated overnight at 4°C with a mouse monoclonal anti-BrdU antibody (1:200; Becton Dickinson). For chromogenic immunodetection, sections were then incubated for 1 h in biotin-conjugated species-specific secondary antibodies (1:100; Vector Laboratories) followed by a peroxidase-avidin complex solution according to the manufacturer’s protocol. The peroxidase activity of immune complexes was visualized with DAB staining using the VectaStain ABC kit (Vector Laboratories). For fluorescent double labeling, that were performed to determine the cell phenotype, sections were incubated overnight at 4°C with a mouse monoclonal anti-BrdU antibody (1:200; Becton Dickinson), and a goat anti-DCX (1:200, Santa Cruz Laboratories). Antibodies were revealed with anti-mouse Alexa 488 and anti-goat Alexa 594 coupled antibodies (1:400; Molecular Probes). All BrdU-labeled cells in the granular cell layer and subgranular zone (SGZ) were counted in each section (*n* = 10 and 5 mice per group) at 400× magnification under a light microscope (Olympus) by an experimenter blinded to the study code. The total number of BrdU-positive cells per section was multiplied by 6 to obtain the total number of cells per dentate gyrus. The counting of BrdU/DCX labeled cells was performed using a Laser Scanning Confocal Microscope (TCS SP, Leica) equipped with a DMIRBE inverted microscope.

### Extracellular Unitary Recordings of DRN 5-HT Neurons

Single-barreled glass micropipettes (recording electrodes) were filled with a 2 M NaCl solution saturated with Fast Green FCF, resulting in an impedance of 2–5 MΩ. Mice were anesthetized with chloral hydrate (400 mg/kg, using a 2% solution), and placed in a stereotaxic frame equipped with the Stoelting mouse adapter. Electrodes were positioned 0.5–1 mm posterior to the interaural line on the midline, and were then lowered into the DRN at a depth of 2.5 mm from the brain surface. 5-HT neurons were then encountered over a maximal distance of 1 mm. They were identified using the following criteria: a slow (0.5–2.5 Hz) and regular firing rate and long-duration (0.8–1.2 ms) action potentials (Heurteaux et al., 2006). Spikes were computed by using the Spike 2 software. Firing rates were calculated as the mean number of events occurring within a 10 s period. For each neuron, discharges were monitored during 60 s. Three to four successive descents were performed along the DRN, for a total of 4–8 cells recorded per animal. Recordings were performed for a maximal duration of 4 h post-injection.

## Results

### *Sort1*^−/−^ Mice Behavior in Depression and Anxiety Tests

In the aim to confirm the role of sortilin on the TREK-1 functions in depression, we first investigated the behavior of *Sort1*^−/−^ mice in a series of behavioral tests related to depression and anxiety.

#### Depression Tests

**FIGURE 1:**
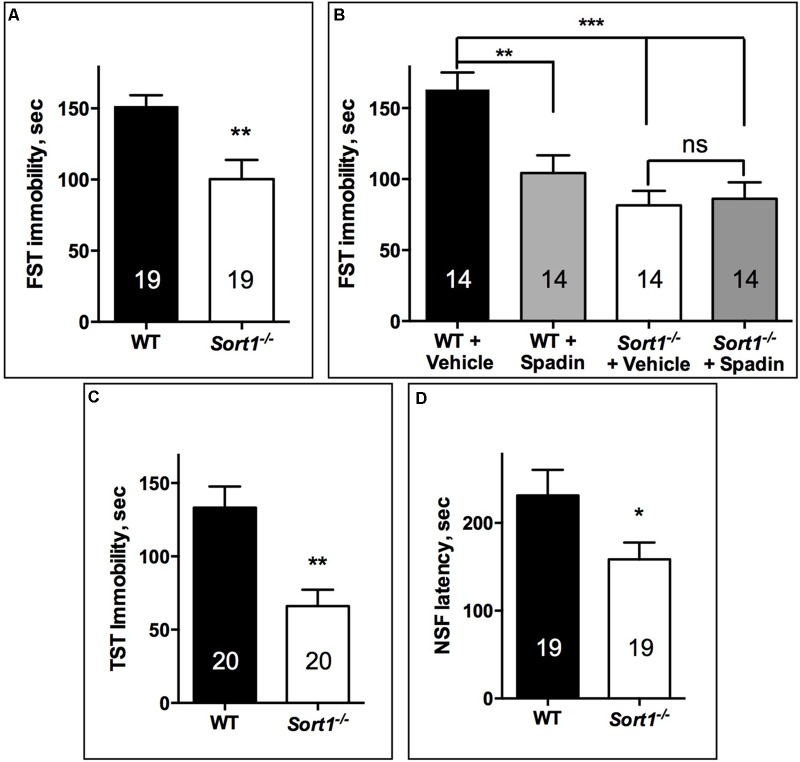
Antidepressant-like behavior in *Sort1*^−/−^ mice. **(A)** FST, *Sort1*^−/−^ mice had a shorter immobility time than WT mice. **(B)** WT spadin-treated mice had also a shorter immobility time than vehicle-treated mice. Spadin had no effect on *Sort1*^−/−^ mice. **(C)** TST, *Sort1*^−/−^ mice had a significant reduced immobility time than WT mice. **(D)** NSF, the latency time for *Sort1*^−/−^ mice was shorter than that measured for WT mice. Values are expressed as mean ± SEM. ^∗^*p* < 0.05, ^∗∗^*p* < 0.01, ^∗∗∗^*p* < 0.001.

The FST performed directly on both WT and *Sort1*^−/−^ mouse groups indicated that *Sort1*^−/−^ mice presented a significant lower immobility time (100.3 ± 13.5 s) than WT mice (151.4 ± 7.8 s, *p* = 0.0041) (**Figure 1A**). FST was validated on WT mice by the use of a novel potential AD spadin validated at least in rodents (Mazella et al., 2010) (10 μg/kg) which after i.p. injection decreased the immobility time from 162.9 ± 12 s for vehicle injected to 104.4 ± 12.5 s (*p* = 0.0045) (**Figure 1B**). We also confirmed the effect of genotype since vehicle injected *Sort1*^−/−^ mice presented a significant lower immobility time (81.6 ± 10.1 s) than WT vehicle injected (162.9 ± 12 s, *p* < 0.001) [two-way ANOVA, *F*(3,39) = 10.69, *p* < 0.001] (**Figure 1B**). Interestingly, injection of spadin had no effect on *Sort1*^−/−^ mice (86.1 ± 11.6 s, *p* = 0.992) (**Figure 1B**). This result prompted us to perform two other tests, the TST and the NSF test. The TST confirmed that *Sort1*^−/−^ mice had an immobility time (65.9 ± 11.2 s) significantly reduced when compared to WT mice (133.2 ± 14.4 s, *p* = 0.0013) (**Figure 1C**). Finally, in the NSF test, the latency time measured for WT mice (231.4 ± 29.2 s) was also significantly decreased in *Sort1*^−/−^ mice (158.5 ± 19.1 s, *p* = 0.043) (**Figure 1D**).

#### Anxiety Tests

**FIGURE 2:**
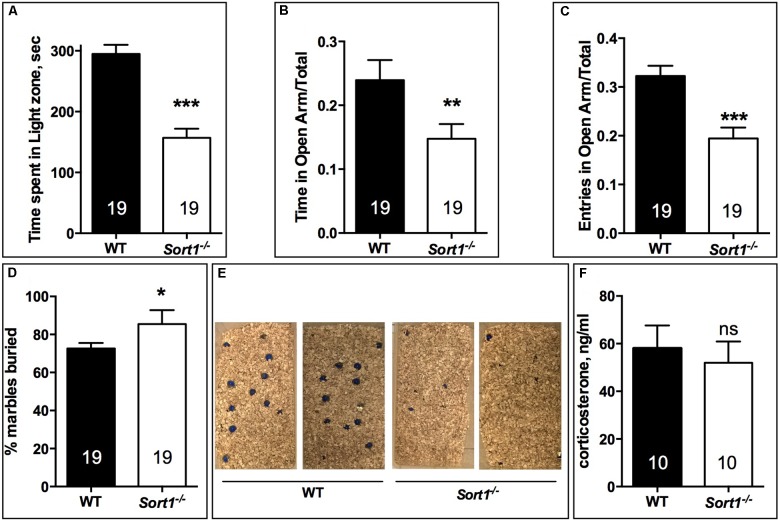
Anxiety-like behavior in *Sort1*^−/−^ mice. **(A)** Light-dark test, the time spent in the light zone was shorter for *Sort1*^−/−^ mice compared to WT mice. **(B,C)** Elevated-plus Maze test, the ratio of the time spent in the open arm was reduced in *Sort1*^−/−^ mice compared to WT mice. The ratio in the number of entries was also reduced in *Sort1*^−/−^ mice. **(D,E)** Glass marbles burying test, the percent of marbles buried by WT mice was significantly increased by *Sort1*^−/−^ mice as shown in **E**. **(F)** Serum corticosterone level in WT and *Sort1*^−/−^ mice remained unchanged. Values are expressed as mean ± SEM. ^∗^*p* < 0.05, ^∗∗^*p* < 0.01, ^∗∗∗^*p* < 0.001, ns, non-significant.

In the light dark test, the time spent in the light zone for WT mice was 294.9 ± 14.9 s, time significantly higher than that of *Sort1*^−/−^ mice (156.9 ± 15.1 s, *p* < 0.001) (**Figure 2A**). Therefore, we tested the two groups in the elevated-plus maze test by measuring the ratio of the time and the number of entries in the open arm. The time ratio in the open arm of WT mice (0.239 ± 0.031) was significantly reduced in *Sort1*^−/−^ mice (0.148 ± 0.023, *p* = 0.0082) (**Figure 2B**). In the same way the number of entries ratio of WT mice (0.322 ± 0.021) was decreased in *Sort1*^−/−^ mice (0.194 ± 0.022, *p* = 0.0003) (**Figure 2C**). Finally, we tested the ability of WT and *Sort1*^−/−^ mice to bury glass marbles, a test able to detect anxiety and obsessive compulsive disorders related phenotypes (Deacon, 2006). **Figures 2D,E** showed that WT mice were able to bury 72.7 ± 2.9% of marbles whereas *Sort1*^−/−^ mice buried 85.5 ± 7.3% (*p* = 0.0131) of marbles suggesting again an anxiety-like behavior of *Sort1*^−/−^ mice as previously observed (Ruan et al., 2016). The serum concentration of corticosterone in basal conditions remained unchanged between WT and *Sort1*^−/−^ mice (**Figure 2F**, *p* = 0.642) indicating that the anxiety-like behavior of *Sort1*^−/−^ mice was not the consequence of the hormone increase.

### Depression Resistant Phenotype of *Sort1*^−/−^ Mice and Modification of TREK-1 Membrane Expression and Function

**FIGURE 3:**
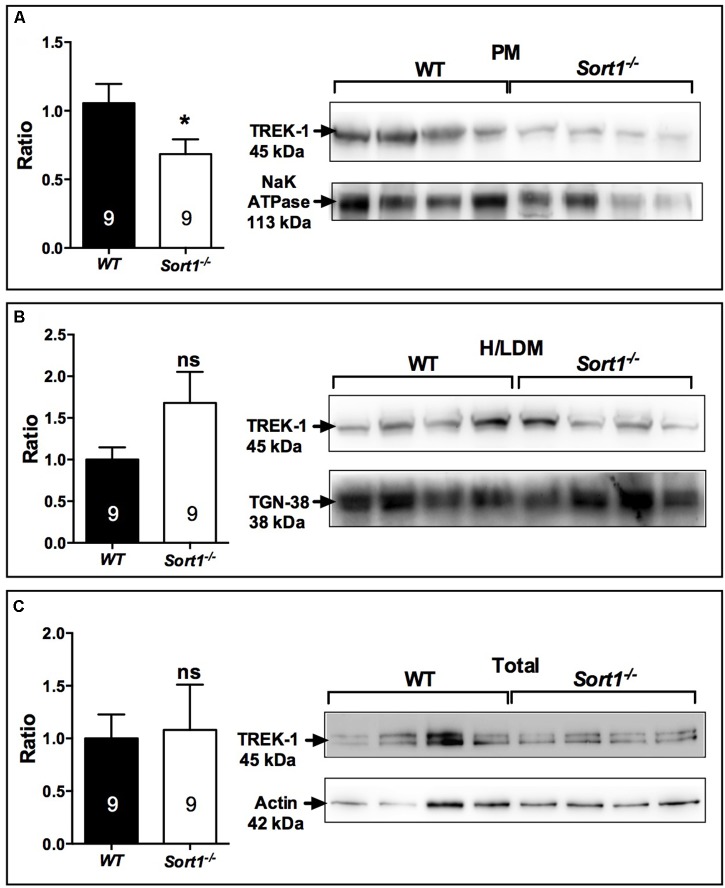
Sub-cellular location of the TREK-1 channel protein in the brain of *Sort1*^−/−^ and WT mice. **(A)** The TREK-1 expression was decreased by 36% (histogram, Left) in the plasma membrane (PM) prepared from *Sort1*^−/−^ mouse brain when compared to PM prepared from WT mouse brain as visualized by Western blots (Right). **(B)** The expression of TREK-1 channels remained unchanged in high and low density vesicles (H/LDM) prepared from brains of *Sort1*^−/−^ and WT mice, as well as in total brain extracts **(C)**. The sub-cellular compartments were identified by specific markers: NaKATPase for plasma membranes **(A)**, TGN38 for H/LDM **(B)**, and actin for total brain extracts **(C)**. ^∗^*p* < 0.05, ns, non-significant.

The decrease of depressive-like behavior of *Sort1*^−/−^ mice may suggest some dysfunctions of the TREK-1 channel for which its blockade or its deletion results in a depression-resistant phenotype. We first investigated the subcellular location of TREK-1 performed on sub-cellular fractionation from WT and *Sort1*^−/−^ mouse brains. Surprisingly, the amount of TREK-1 channels present at the PM was significantly decreased by 36% in *Sort1*^−/−^ mice (*p* = 0.0376) when compared to WT mice (**Figure 3A**). The loss of TREK-1 at the PM appeared to be compensated by an increase of the protein at the level of high and low density vesicles (H/LDM) although not significant (**Figure 3B**, *p* = 0.159). The total amount of TREK-1 was not modified between WT and *Sort1*^−/−^ mice (**Figure 3C**, *p* = 0.776). The loss of the TREK-1 channels at the cell surface without modification of total TREK-1 expression is in agreement with the role of sortilin in the channel cell sorting as already observed (Mazella et al., 2010).

**FIGURE 4:**
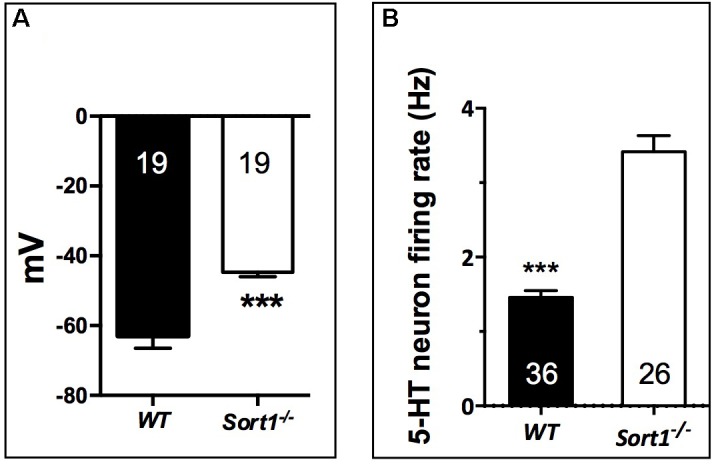
Neuronal activity in *Sort1*^−/−^ mice. **(A)** Membrane potential mean values obtained on primary cortical neurons prepared from *Sort1*^−/−^ and WT mice showed an important depolarization in *Sort1*^−/−^ neurons when compared with WT neurons (ΔmV = 18.21 mV). **(B)** Dorsal Raphe Nucleus 5-HT neuronal firing activity recorded from WT and *Sort1*^−/−^ mice. ^∗∗∗^*p* < 0.001.

To determine the consequence of the TREK-1 expression loss at the PM, we performed electrophysiological experiments on neurons prepared from WT and *Sort1*^−/−^ mice. The membrane potential measured on neurons from *Sort1*^−/−^ mice was 44.7 ± 1.3 mV consisting to a significant increase of membrane potential of 18.21 mV (*p* = 0.0003) when compared to neurons from WT mice (62.9 ± 3.6 mV) (**Figure 4A**). This result indicated that the decrease of TREK-1 channels at the cell surface of *Sort1*^−/−^ neurons leads to an increase of membrane potential very similar to that obtained with the channel blocker spadin on WT neurons (Devader et al., 2015).

Previous studies have shown that the blockade (Mazella et al., 2010) or the TREK-1 deletion (Heurteaux et al., 2006) enhances the midbrain 5-HT neuron firing rate, a key parameter predictive of AD efficacy. Therefore, we performed unitary extracellular recordings of 5-HT neurons in the DRN in anesthetized animals. The basal firing activities were recorded in both WT and *Sort1*^−/−^ mice by successive tracks along the DRN. 5-HT neurons found in WT mice discharged within a normal frequency range (1.5–2 Hz) with a twofold lower average than in *Sort1*^−/−^ mice (1.46 ± 0.09 Hz versus 3.42 ± 0.21 Hz, *p* < 0.001, *t* = 9.21, *df* = 60) (**Figure 4B**).

**FIGURE 5:**
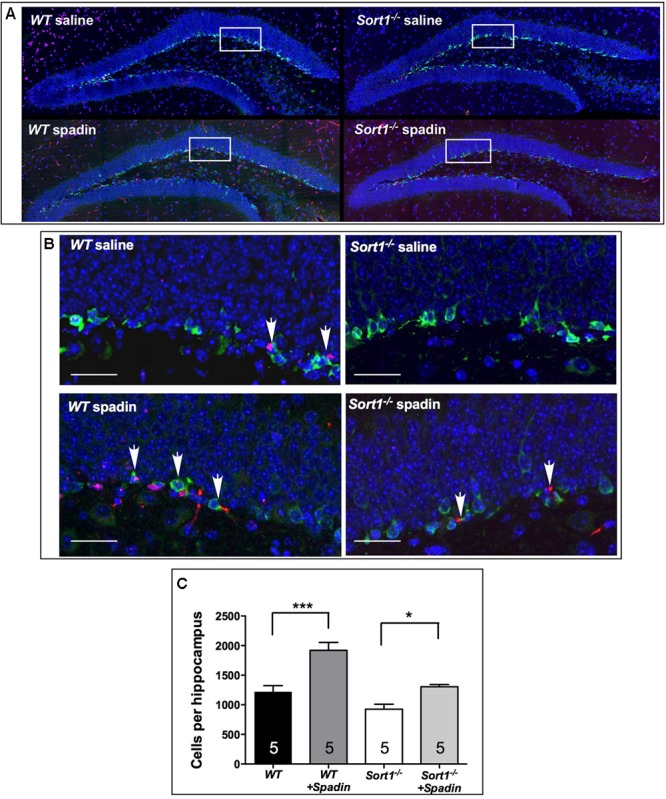
Neurogenesis in *Sort1*^−/−^ mice. **(A)** BrdU staining of dentate gyrus slices from WT and *Sort1*^−/−^ mice treated with saline or spadin for 4 days. **(B)** Double labeling of BrdU-labeled neurons with DCX (neuronal precursor marker), showing a colocalization in magnificated regions, bar: 50 μm. **(C)** 4 Days spadin treatment increased the number of BrdU cells in hippocampus from WT and *Sort1*^−/−^ mice (*n* = 5 per genotype), expressed as mean ± SEM. ^∗∗∗^*p* < 0.001 versus vehicle injected WT mice and ^∗^*p* < 0.05 versus vehicle-injected *Sort1*^−/−^ mice (vehicle-or-spadin-injected, Tukey’s multiple comparison test).

It is established that chronic AD treatments, including fluoxetine but also the fast-acting AD spadin induce neurogenesis in the hippocampus of rodents visualized by an increase of progenitor cells that incorporate BrdU. WT mice treated with spadin (100 μg per kg body weight, ip) for 4 days showed a significant increase in the number of hippocampal BrdU-positive cells compared with WT mice treated with vehicle [1919 ± 134 cells/hippocampus vs. 1218 ± 105 cells/hippocampus, respectively, two-way ANOVA, *F*(3,12) = 31.36, *p* < 0.001] (**Figure 5C**). In *Sort1*^−/−^ mice, although the number of BrdU-positive cells was slightly lower in basal conditions (*p* = 0.113), spadin remained efficient with a lower but significant increase of hippocampal newborn cells from 927 ± 83 in WT to 1306 ± 37 in *Sort1*^−/−^ hippocampus, respectively (*p* = 0.0331) (**Figure 5**).

### Constitutive Activation of BDNF Content in *Sort1*^−/−^ Mice

**FIGURE 6:**
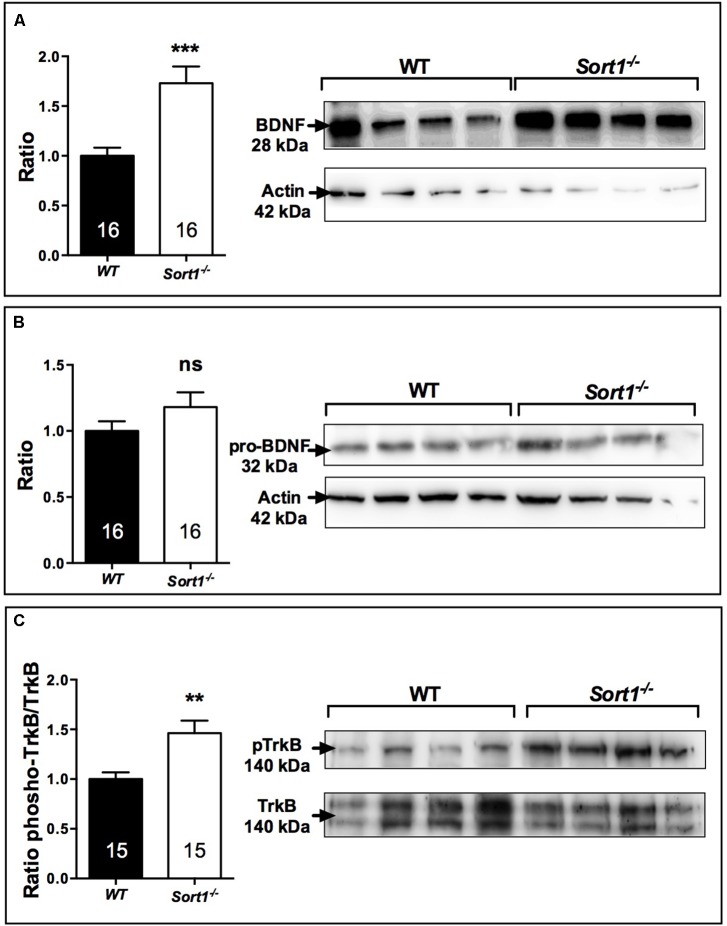
Effect of deletion of *Sort1*^−/−^ gene on BDNF system. Western blot analyses and their corresponding histogram quantification of the expression of proteins involved in the BDNF system in brain extracts from WT and *Sort1*^−/−^ mice. **(A)** BDNF, **(B)** proBDNF, and **(C)** phospho-TrkB. ^∗∗^*p* < 0.01, ^∗∗∗^ *p* < 0.001, ns, non-significant.

Brain derived neurotrophic factor displays a potential role as a marker of treatment response in patients with major depressive disorder although its effects on mood variations remain unclear. The release of BDNF from the neuronal regulatory pathway has been shown to be dependent on the presence of sortilin (Chen et al., 2005). For these reasons and because *Sort1*^−/−^ mice displayed a depressive resistant phenotype, we evaluated the expression profiles of BDNF and its precursor proBDNF in the brain of WT and *Sort1*^−/−^ mice. Western blot analyses of BDNF content from WT or *Sort1*^−/−^ mouse brains clearly demonstrated that the neurotrophin amount was strongly increased in *Sort1*^−/−^ mice versus WT (*p* = 0.0005) (**Figure 6A**). By contrast, no change in amount of proBDNF was observed (**Figure 6B**, *p* = 0.441). The BDNF increase prompted us to examine the expression of the active phosphorylated form of the BDNF receptor TrkB. The p-TrkB expression was significantly enhanced in *Sort1*^−/−^ (*p* = 0.0023) mice when compared with WT mouse brain (**Figure 6C**).

**FIGURE 7:**
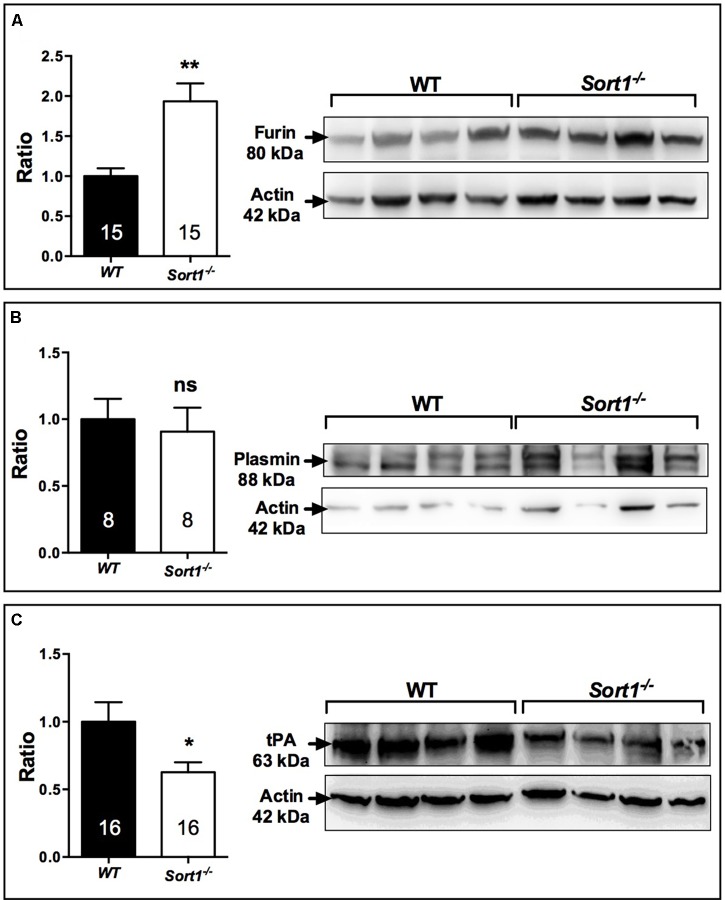
Effect of deletion of *Sort1*^−/−^ gene on BDNF releasing pathways. Western blot analyses and their corresponding histogram quantification of the expression of proteins involved in the BDNF releasing pathways in brain extracts from WT and *Sort1*^−/−^ mice. **(A)** Furin, **(B)** Plasmin, and **(C)** tPA. Histograms represented ratio of mean ± SEM of protein quantification from indicated number of brain samples. Mann–Whitney test, ^∗^*p* < 0.05; ^∗∗^*p* < 0.01, ns, non-significant.

To determine which of BDNF secretion pathway was affected in *Sort1*^−/−^ mice we examined both the expression of furin, responsible for the intracellular maturation of proBDNF to the constitutive secretion pathway, and the expression of the complex plasmin/tPA, responsible for the extracellular maturation of the regulated proBDNF secretion (Lu et al., 2005). Interestingly, in *Sort1*^−/−^ mice, the brain expression of furin was strongly enhanced (*p* = 0.0014) (**Figure 7A**) whereas the expression of tPA, the plasmin activator, was significantly decreased in *Sort1*^−/−^ mice (*p* = 0.0385) (**Figure 7B**). Plasmin expression was not modified (*p* = 0.56) (**Figure 7C**).

The increase in the expression of BDNF and its activated receptor TrkB observed in *Sort1*^−/−^ mice could suggest a constitutive activity of the BDNF signaling system.

## Discussion

The present work shows for the first time that the cellular localization and function of the TREK-1 channel were altered in mice lacking sortilin. The TREK-1 expression decrease within the brain PM of *Sort1*^−/−^ mice compared to WT mice (**Figures 3A–C**) highly suggests a crucial role of sortilin in the correct sorting of the TREK-1 channel. Further quantification of immunocytochemical labeling of TREK-1 could improve demonstration of its decreased PM expression but specific TREK-1 antibodies are not yet available for such studies. The involvement of sortilin in the TREK-1 trafficking has previously been documented in a heterologous expression system by cotransfection of the two proteins (Mazella et al., 2010). TREK-1 is not the only membrane protein that is regulated by an interaction with sortilin. In particular, sortilin displays important action in the function of several receptors including the proNGF receptor p75NTR (Nykjaer et al., 2004), the BDNF receptor TrkB (Yang et al., 2011), the neurotensin receptor NTSR1 (Martin et al., 2002), and NTSR2 (Beraud-Dufour et al., 2009). In the present work, the absence of sortilin allowed us to definitively determine that either the TREK-1 dysfunction by alteration of its sorting or its inhibition by spadin leads to a common result: the resistance to depression behavior. This phenotype is confirmed in the three different behavioral tests performed in *Sort1*^−/−^ mice (**Figure 1**). Taken together, these results indicated that *Sort1*^−/−^ mice appear to display a depression-resistant behavior resembling that of *kcnk2*^−/−^ mice (Heurteaux et al., 2006). Indeed, this phenotype was comparable to that of *Kcnk2*^−/−^ mice (lacking TREK-1) which behaved as wild-type mice treated with classical ADs. However and paradoxically, *Sort1*^−/−^ mice displayed an anxiety-like behavior in several anxiety-related tests including the light dark and the marbles burying tests (**Figure 2**). This latter observation that appears not to be associated with an elevated level of serum corticosterone, is in agreement with a recent study relating that *Sort1*^−/−^ mice displayed elevated anxiety-like behavior and that chronically stressed wild-type mice showed an increase in the sortilin expression in neocortex and hippocampus leading to an increased depression-like behavior (Ruan et al., 2016). Since the increase of sortilin expression is associated with depression, our results describing a resistance to depression in *Sort1*^−/−^ mice could be the consequence of the absence of sortilin.

The neuronal consequences resulting from the decreased cell surface TREK-1 expression led to several observations: (i) the membrane potential measured on *Sort1*^−/−^ neurons was strongly increased when compared to WT neurons (**Figure 4A**), and (ii) the firing rate activity of neurons from the DRN was twofold increased indicating an accelerated efficacy of the DRN neurotransmission which could be attributed to 5-HT neurotransmission (**Figure 4B**) although further additional pharmacological and microdialysis approaches have to be performed to definitively identify 5-HT neurotransmission. Both observations were very similar to those obtained with the blocking of TREK-1 activity by spadin (Mazella et al., 2010; Devader et al., 2015) and by fluoxetine or when kcnk2 gene was deleted in TREK-1 null mice (Heurteaux et al., 2006). These properties are in good agreement with the observation that pharmacological and electroconvulsive AD treatments enhance activation of hippocampal post-synaptic 5-HT_1A_ receptors (Haddjeri et al., 1998).

While the number of hippocampal progenitor cells remained unchanged between *Sort1*^−/−^ and WT mice, surprisingly, both strains responded, similarly, to a 4 days spadin treatment with a significant increase in the number of BrdU-positive cells. This result suggests that (1) despite the lower level of TREK-1 membrane expression measured in *Sort1*^−/−^ mice, the remaining functional channels are sufficient to trigger neurogenesis under spadin exposure (note that the response to spadin was quite lower in *Sort1*^−/−^ mice) or (2) TREK-1 is not the only membrane protein responsible for the response to the peptide. In neurogenesis experiments *Sort1*^−/−^ mice were still able to respond to spadin (**Figure 5C**). Similar observations were obtained from *kcnk2*^−/−^ mice in which basal hippocampal neurogenesis was identical to that measured from WT mice but *kcnk2*^−/−^ mice remained able to respond to fluoxetine (Heurteaux et al., 2006). In *Sort1*^−/−^ mice, spadin was unable to display a significant behavioral effect in FST but was able to increase neurogenesis. This can be explained by methodological differences between the two types of experiments, for FST, spadin was injected 30 min before the test whereas neurogenesis quantification was performed after a spadin treatment for 4 days. This could correspond to the difference of effects between an acute effect (FST) and a semi-chronic effect (neurogenesis) for spadin. The other possible interpretation could be that the signaling pathways involved in neurogenesis are partly distinct in *Sort1*^−/−^ and *kcnk2*^−/−^ mice.

Finally, we observed that the level of brain BDNF, as well as its activated receptor TrkB, was significantly increased in *Sort1*^−/−^ mice, whereas the level of its precursor form proBDNF remained unchanged (**Figures 6A–C**). This finding can be explained by the brain over-expression in *Sort1*^−/−^ mice of the convertase furin involved in the regulated pathway of BDNF secretion (**Figure 6A**). Concomitantly, the plasmin and tPA expression involved in the constitutive secretion pathway, appeared slightly decreased (**Figures 6B,C**). The increase in both BDNF secretion and expression has been already observed in *Sort1*^−/−^ neurons (Chen et al., 2005) that have been prepared slightly differently than those used in this study (Devader et al., 2016). The elevated levels of BDNF and of its activated receptor TrkB in the *Sort1*^−/−^ brains could be responsible for the antidepressive-like behavior although we cannot compare data from neurogenesis measured in the hippocampus with the level of BDNF measured from the whole brain.

The limitations of this study concern first the fact that only male were used in this work. Further experiments with females as well as more detailed characterization of signaling pathways involved in the observed effects will be necessary.

## Conclusion

In conclusion, data presented in this work could explain the molecular and physiological mechanisms that are responsible for the decrease of depressive-like behavior observed when the expression of TREK-1 was decreased or totally absent.

## Author Contributions

SM, CD, MP, and JM performed the experiments. MB, CH, and JM conceived and designed the experiments, contributed reagents, materials, and analysis tools, and wrote the paper.

## Conflict of Interest Statement

The authors declare that the research was conducted in the absence of any commercial or financial relationships that could be construed as a potential conflict of interest.

## Abbreviations

5-HT: serotonin
AD: antidepressant
BDNF: brain derived neurotrophic factor
BrdU: 5-bromo-2′-deoxyuridine
DCX: doublecortin
DRN: dorsal raphe nucleus
FST: forced swimming test
H/LDM: high and low density microsomes
NSF: novelty suppressed feeding
NT: neurotensin
NTSR2: neurotensin receptor-2
NTSR3: neurotensin receptor-3
p75NTR: neurotrophin receptor of 75 kDa
PM: plasma membrane
TGN38: trans-golgi network protein of 38 kDa
TrkB: tropomyosin receptor kinase B
TST: tail suspension test
WT: wild type
